# Verrucous venous malformation: A case report of a rare entity with dermatoscopic features

**DOI:** 10.1016/j.jdcr.2024.10.022

**Published:** 2024-11-10

**Authors:** Amal Hani Abualola, Waseem Alhawsawi, Sahal Samarkandy, Hatim AlMaghrabi

**Affiliations:** a College of Medicine, King Saud bin Abdulaziz University for Health Sciences, Jeddah, Saudi Arabia; b Dermatology Department, King Abdulaziz Medical City, National Guards Affairs, Jeddah, Saudi Arabia; c Dermatology Department, King Fahad Armed Forces Hospital, Jeddah, Saudi Arabia; d Pathology Department, King Faisal Specialist Hospital and Research Centre, Al-Madinah Al-Munawwarah, Saudi Arabia

**Keywords:** dermatoscopy, verrucous hemangioma, verrucous venous malformation

## Introduction

Verrucous venous malformation (VVM), formerly known as verrucous hemangioma, is a rare type of congenital vascular malformations that typically presents as a raised, warty lesion on the skin. It was first described in 1991 by Mulliken and Glowacki, who coined the term “verrucous hemangioma” to describe the distinctive clinical and histopathologic features of the lesion. The exact cause of VVM is not known, but it is thought to be a developmental defect in the formation of blood vessels. It is not inherited and typically appears sporadically, although there have been rare reports of familial cases. Somatic missense mutations in the mitogen-activated protein kinase kinase kinase 3 gene were detected in 6 of 10 VVMs. There are no sexual, racial, or ethnic predilections. It typically appears during early childhood but can develop later in life.^1^^,^^2^ Most of the time, it appears in early life as nonkeratotic reddish macular area resembling infantile hemangioma precursor, however, VVM has a distinct nature as it increases in size, spreads locally, and becomes warty, bluish, and hyperkeratotic later in life.^1^, ^2^, ^3^ VVM is usually located in the lower limb of children, being approximately 95% of the cases unilaterally.^4^ Moreover, VVM lesions show no tendency to regress or resolve spontaneously.^2^ It can be misdiagnosed as angiokeratoma, venous or lymphatic malformation, or infantile hemangioma clinically, so histopathologic and dermatoscopic evaluation could help in differentiating between them. Histopathologiclly, VVM is characterized by dilated venules and capillaries in the papillary dermis, which are surrounded by proliferating epidermis and hyperkeratosis. The lesion can occur anywhere on the body, but is most commonly found on the extremities, particularly the lower portion of the legs. Diagnosis of VVM is based on clinical and histopathologic findings.^5^ Treatment for verrucous hemangioma is often challenging, as the lesion can be resistant to conventional therapies such as laser treatment or surgical excision. Treatment with pulsed dye laser has being reported to be of variable results. Eg, a 13-year-old boy with VVM showed significant improvement in symptoms and lesion size after 3 years of sirolimus treatment with no side effects have been reported.^6^ Some cases have been treated successfully with topical imiquimod or intralesional injection of bleomycin or steroids, although further research is needed to assess the effectiveness of these treatment.^5^

## Case presentation

**Fig 1:**
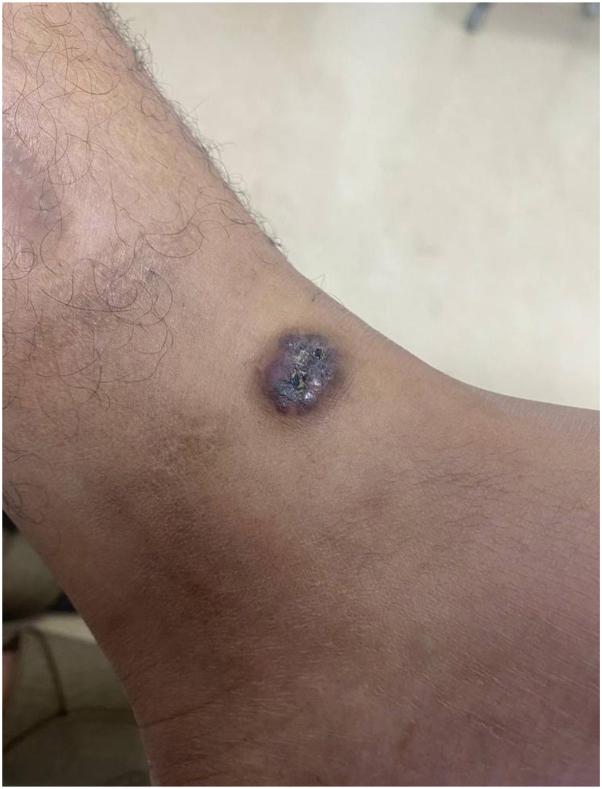
Gross appearance of verrucas nodule on the left ankle.

**Fig 2:**
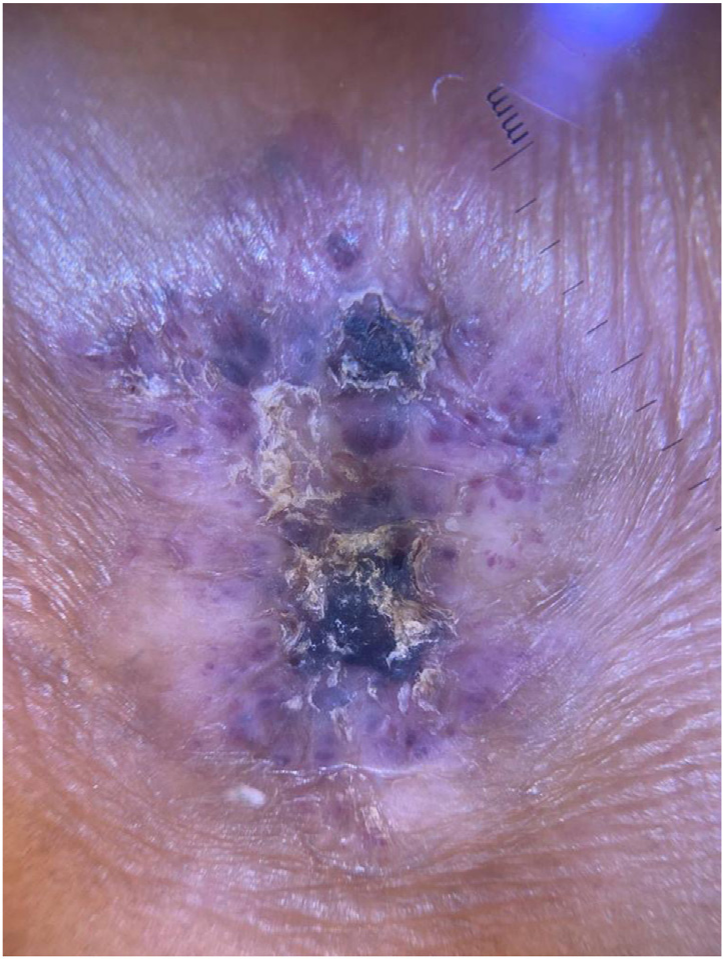
An image of the lesion showing dermatoscopy characteristics.

**Fig 3:**
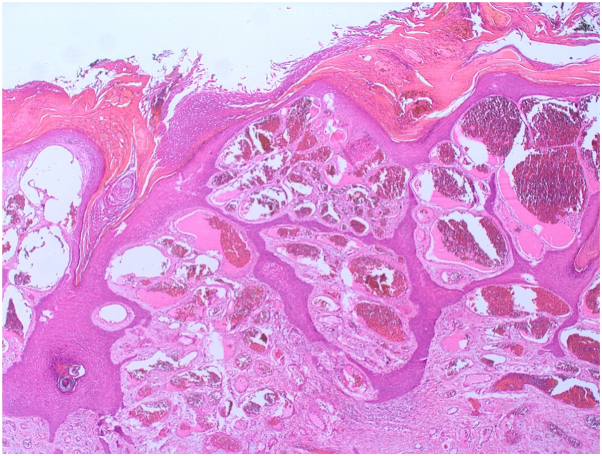
Digital photomicrograph reveals, from top to bottom: hyperkeratosis, irregular papillomatosis with acanthosis. Multiple dermal small and ectatic vascular spaces, some of which appear to be encircled squamous cells. (Hematoxylin-eosin stain; original magnification: ×40.)

**Fig 4:**
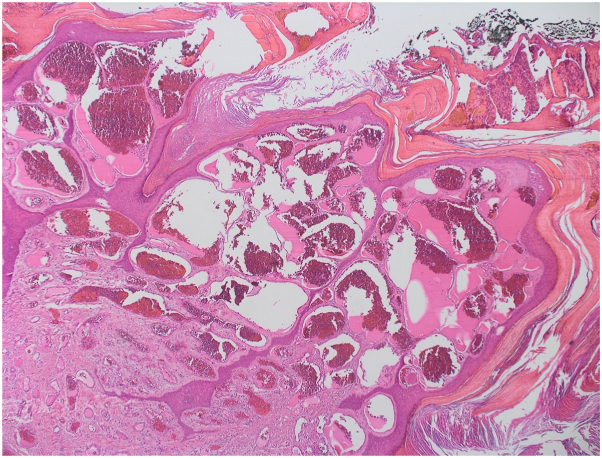
Digital photomicrographs reveal involvement of papillary and reticular dermis. The dermis is expanded by a vascular lesion comprised of variable size ectatic vascular spaces forming lakes of blood. (Hematoxylin-eosin stain; original magnification: ×400.)

This is a 29-year-old man presented to our clinic complaining of solitary skin nodule over the lateral side of the ankle since preschool ages. The lesion tends to enlarge slowly with changes in architecture with no tendency toward regression. The lesion is asymptomatic, however, easily gets ulcerated and irritated, hence bleeding with traumatization as the patient is a soccer player. The lesion does not extend to other skin areas. The patient is not known to have any medical illnesses nor past surgical history. No similar cases in the family. The patient tried multiple excision attempts in other clinics, but recurrences led him to our clinic. Physicals examination showed solitary, blanchable, and violaceous verrucous hyperkeratotic nodule over the lateral side of the left malleolus (Fig 1). Dermatoscopic examination (Fig 2) showed asymmetric purplish globules that are variable in size and color intensity. Darker and larger globules are located centrally, whereas smaller and brighter globules were clustered peripherally in alveolar pattern. Secondary hyperkeratosis and crustation were evident, in addition to areas of fibrosis surrounding the whole lesion. Excisional biopsy showed hyperkeratosis with ecstatic superficial and deep venulocapillaries. As demonstrated in Figure 3 digital photomicrograph reveals, from top to bottom: hyperkeratosis, irregular papillomatosis with acanthosis. Multiple dermal small and ectatic vascular spaces, some of which appear to be encircled squamous cells. In addition, digital photomicrographs in Figure 4 reveals involvement of papillary and reticular dermis. The dermis is expanded by a vascular lesion comprised of variable size ectatic vascular spaces forming lakes of blood. Given the above clinicopathologic inputs, a diagnosis of VVM was made. The patient was treated with pulsed dye laser sessions with a good initial response on the first follow-up.

## Discussion

VVM is a rare vascular entity characterized by malformation of cutaneous capillaries and venules that is thought to be caused by genetic alterations in mitogen-activated protein kinase kinase kinase 3 enzyme. Morphologically, VVM closely resembles angiokeratoma, which is a reactive papillary dermal tumor, or malformation in angiokeratoma circumscriptum variant. In this case, we focused on the dermatoscopic features since only 4 cases of VVM with dermatoscopic features have been reported as demonstrated in Table I.^5^^,^^7^, ^8^, ^9^ The focus on dermatoscopy in this era significantly aided in establishing differential diagnoses. Angiokeratoma dermatoscopic features include lacunae with varying shades of red, maroon, and blue. There may be thrombosed lacunae displaying a black color. The central portion of the lesion often has a blue-whitish veil, and the periphery often has an erythematous halo. On the basis of dermatoscopy, angiokeratoma, and VVM cannot be distinguished easily via dermatoscope. Angiokeratoma tends to have larger, monomorphic, and darker lacunae. Whereas VVM tends to have smaller, variable size, and brighter globules dermatoscopically. Hence, dermatoscopy can aid in diagnosing such cases but it still cannot be decisive as there are overlap between its features. As in Table II that shows dermatoscopic findings of verrucous hemangioma and its differential diagnosis.^5^

**Table I:** Comparison of dermatoscopic feature of cases reported VVM

Verrucous venous malformation cases	Authors	Dermatoscopic features
Verrucous hemangioma (also known as verrucous venous malformation): a vascular anomaly frequently misdiagnosed as a lymphatic malformation	Boccara et al^7^	Vascular pattern of nonkeratotic areas characterized by an erythematous patch scattered with little vascular red to violet dots
Linear verrucous hemangioma—a rare case and dermatoscopic clues to diagnosis	Dhanta et al^5^	Prominent hyperkeratosis over bluish background along with the reddish blue lacunae, indicating underlying dilated vascular channels. Peripheral areas of the lesion showing the bluish lacunae
A case of verrucous hemangioma and its dermatoscopic features	Prabhakar and Kaliyadan^8^	Early lesions of verrucous hemangioma show a prominent bluish white hue, indicating the hyperkeratosis over the underlying vascular channels Peripheral areas of the lesion show the dark blue lacunae characteristic of vascular lesions Late lesions showing the more prominent hyperkeratosis along with the bluish lacunae indicating the underlying dilated vascular channels
Evolution of verrucous hemangioma	Popadic^9^	Alveolar appearance, numerous small, oval, and polygonal elements surrounded by slightly darker pigmentation and mildly presented sulci. Purplish-brown color is the predominant pigmentation. Typical lacunae of varying dimensions found on the lower half of the lesion margin

**Table II:** Dermatoscopic findings of verrucous hemangioma and its differential diagnosis^5^

1	Verrucous hemangioma	Alveolar appearance with various shadows of bluish small, oval to polygonal elements surrounded by slightly darker pigmentation with well-defined dark lacunae in the periphery. Dominant hyperkeratosis seen in the verrucous lesions
2	Infantile hemangioma	Polymorphous pattern of vascular structures with or without red linear and red dilated vessels
3	Angiokeratoma	Dark lacunae and whitish veil, peripheral erythema, and hemorrhagic crust in third pattern
4	Pigmented basal cell carcinoma	Leaf-like and spoke-wheel pigmentation, arborizing vessels, erosions, blurred lacunae that may look like blue-gray ovoid nests
5	Verrucous epidermal nevus	Large brown circle seen as oval or round structures with a hyperchromic brown edge surrounding a hypochromic area
6	Seborrheic keratosis	Milia-like cysts, comedo-like openings, fissures, and ridges and sharply demarcated border

## Conclusions

Making this benign lesion well recognized by dermatologists using dermatoscopy is significant for the early recognition of VVM. We encourage for future comprehensive researches and documentation of such cases to contribute to identify an in common feature dermatoscopically for possible reduction of invasive procedures as biopsies.

## Conflicts of interest

None disclosed.
